# Carrier Injection and Transport in Blue Phosphorescent Organic Light-Emitting Device with Oxadiazole Host

**DOI:** 10.3390/ijms13067575

**Published:** 2012-06-19

**Authors:** Tien-Lung Chiu, Pei-Yu Lee

**Affiliations:** Department of Photonics Engineering, Yuan Ze University, Taoyuan 32003, Taiwan; E-Mail: st890512@hotmail.com

**Keywords:** phosphorescent OLED, oxadiazole, electron injection, electron transport

## Abstract

In this paper, we investigate the carrier injection and transport characteristics in iridium(III)bis[4,6-(di-fluorophenyl)-pyridinato-N,C2′]picolinate (FIrpic) doped phosphorescent organic light-emitting devices (OLEDs) with oxadiazole (OXD) as the bipolar host material of the emitting layer (EML). When doping Firpic inside the OXD, the driving voltage of OLEDs greatly decreases because FIrpic dopants facilitate electron injection and electron transport from the electron-transporting layer (ETL) into the EML. With increasing dopant concentration, the recombination zone shifts toward the anode side, analyzed with electroluminescence (EL) spectra. Besides, EL redshifts were also observed with increasing driving voltage, which means the electron mobility is more sensitive to the electric field than the hole mobility. To further investigate carrier injection and transport characteristics, FIrpic was intentionally undoped at different positions inside the EML. When FIrpic was undoped close to the ETL, driving voltage increased significantly which proves the dopant-assisted-electron-injection characteristic in this OLED. When the undoped layer is near the electron blocking layer, the driving voltage is only slightly increased, but the current efficiency is greatly reduced because the main recombination zone was undoped. However, non-negligible FIrpic emission is still observed which means the recombination zone penetrates inside the EML due to certain hole-transporting characteristics of the OXD.

## 1. Introduction

Organic light-emitting devices have attracted lots of attention in display and lighting applications due to the various advantages such as self-emission, flexible-substrate compatibility, and large-sized fabrication [1–5]. For efficient use of the triplet exciton for electroluminescence, phosphorescent dopant is employed in the matrix as the emitting layer (EML) of the OLED [6–15]. Contrary to conventional fluorescent dopant materials, dopant concentrations of phosphorescent ones are high due to the short range Dexter energy transfer process [16,17]. This high dopant concentration in phosphorescent OLEDs in turn affects the carrier injection and transport characteristics [18–20]. Due to the better energy level alignments, dopants may help carrier injection by transporting layers into the emitting layer. Some phosphorescent materials are found to exhibit very high carrier mobility comparable to conventional transporting materials [21,22]. On the other hand, sometimes dopant materials can be viewed as trap sites in the EML [23–27]. Overall speaking, in phosphorescent OLEDs, carrier transport should be regarded as two-channel conduction. The carrier may hop through the matrix or dopant sites. Hopping between these two channels is also possible depending on the different dopant concentrations.

Oxadiazoles typically exhibit electron transporting characteristics which can be used as the host for phosphorescent OLEDs [28–31]. In our previous study, we demonstrated an efficient blue phosphorescent OLED consisting of iridium(III)bis[4,6-(di-fluorophenyl)-pyridinato-*N*,C2′]picolinate (FIrpic) doped into 2-phenyl-5-(2′,4′,6′-trimethyl-[1,1′-biphenyl]-4-yl)-1,3,4-oxadiazole (OXD) possessing good electron transporting characteristics and a wide bandgap [32]. In this paper, we investigate the carrier injection and transport characteristics in the EML of such an OLED. With doping FIrpic inside the OXD, the driving voltage is decreased which means the dopants help carrier injection and transport. From EL spectra analysis, it can also be found that the recombination zone shifts toward the anode side. This means the dopant material improves the electron injection and transport capability. When increasing the driving voltage, the relative intensity at longer wavelength of the EL spectra increases and the recombination zone shifts from inside the EML toward the anode side [33,34]. This means: (1) the hole penetrates inside the EML (at low voltage); and (2) electron mobility increases faster than the hole one with increasing voltage. To further understand the electrical properties inside the OLED, we fabricated three devices with part of the EML undoped. The total thickness of the EML is 30 nm, which consists of 10 nm undoped region and 20 nm doped region. The driving voltages of the three OLEDs are higher than in the uniform doped case, which means the dopants are beneficial for voltage reduction in this case. When the undoped layer is near the cathode side, the driving voltage increases significantly, which means the dopant assisted electron injection plays an important role in our device. Although the J-V characteristics are only slightly shifted for the case with the undoped region close to the anode, the current efficiency decreases a lot because there are no dopants inside the main recombination zone. However, there is still observable light emission, which means the hole is transported over the undoped region (pure OXD) and recombines with an electron.

## 2. Results and Discussion

**Figure 1:**
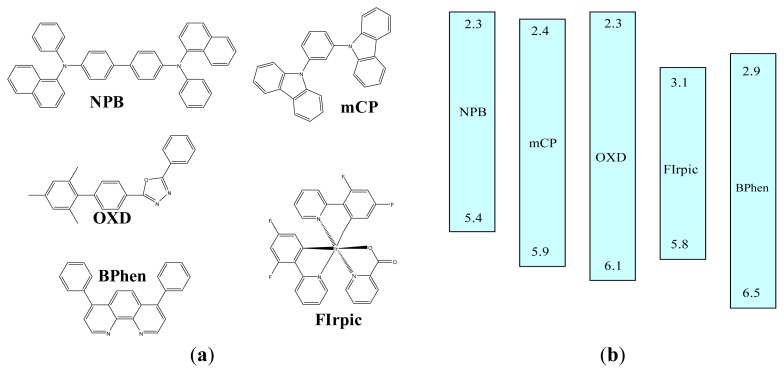
(**a**) Molecular structures of NPB, mCP, OXD, FIrpic, and BPhen; (**b**) Energy levels of all used organic materials.

Table 1 illustrates the layer structures in this study. *N*,*N*′-diphenyl-*N*,*N*′-bis(1-napthyl)-1,1′-biphenyl-4,4′-diamine (NPB), 1,3-bis(carbazol-9-yl)benzene (mCP), 4,7-diphenyl-1,10-phenanthroline (BPhen), LiF, and Al are used as the hole-transporting layer (HTL), exciton blocking layer (EBL), electron-transporting layer (ETL), electron injection layer (EIL), and cathode, respectively. EML is 30 nm with OXD as the host material. The molecular structures and energy levels of these organic materials are shown in Figure 1. For devices 1 to 6, FIrpic concentration increases from 0, 3, 6, 9, 12, to 15%. For devices 7, 8, and 9, the whole EML was separated into three parts, each with 10 nm. For device 7, 10 nm near the ETL side is undoped, while the other 20 nm was doped with 9% FIrpic. For device 8, the center part of the EML is undoped, sandwiched by two doped region. For device 9, 10 nm near the EBL is undoped.

### 2.1. Device Performances of Blue Phosphorescent OLEDs with Different Dopant Concentrations

**Figure 2:**
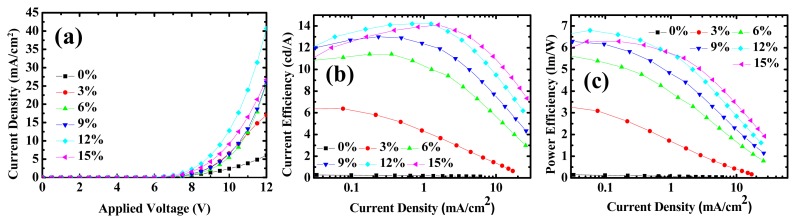
Comparison of (**a**) current density versus voltage; (**b**) current efficiency (cd/A) versus current density; and (**c**) power efficiency (lm/W) versus current density for devices 1 to 6.

Figure 2a shows the J-V characteristics of OLEDs with different dopant concentrations. Compared to the non-doped case (device 1), doping FIrpic (devices 2–6) in the EML helps to reduce the driving voltage, as shown in Table II. Driving voltage is lowest for device 5. Figure 2b,c shows the current efficiency (in terms of cd/A) and power efficiency (in terms of lm/W), respectively. Device 5 also exhibits the highest maximum efficiency. Dopant material inside the matrix plays some role for better conduction, which may be: (1) better hole injection; (2) better hole transport; (3) better electron injection; (4) better electron transport; and (5) higher recombination current. In the following discussion, we will see that better electron injection is the main reason for the voltage reduction, and better electron transport shows only a minor effect.

**Figure 3:**
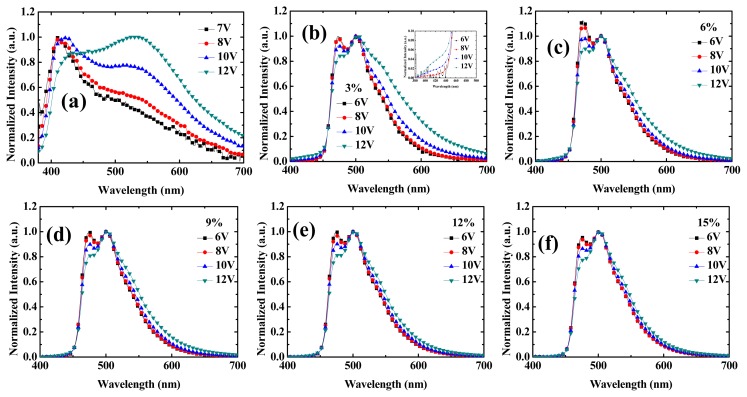
Electroluminescence (EL) spectra at different voltages of devices 1–6 ((**a**–**f**), respectively).

Figure 3 shows the EL spectra for the six devices. For the case of undoped EML (device 1 in Figure 3a), a clear peak at 410 nm originates from mCP emission at lower driving voltage (7 and 8 V). On increasing the driving voltage to 10 and 12 V, this peak redshifts to 420 nm and disappears. On the other hand, the broad exciplex emission due to mCP/OXD interface around 530 nm increases monotonically with increasing driving voltage, which also implies that the recombination zone shifts from inside the OXD to the interface of mCP/OXD. At low driving voltage, the location of the recombination zone inside the OXD can be anticipated because this OXD exhibits certain hole-transporting characteristics. Besides, one can also see an increase around 430 nm with higher driving voltage, which comes from NPB emission, due to the recombination zone shift with increasing driving voltage. The multiple peak spectra in Figure 3a also implies that the recombination zone inside this device is quite broad, and covers NPB, mCP, and OXD. With increasing driving voltage, the recombination zone shifts toward the anode side. This means the electron mobility increases faster than the hole mobility with increasing driving voltage in the mCP and OXD layer. When the OXD is doped with 3% FIrpic (device 2 in Figure 3b), one can see the clear double peak emission at 474 and 502 nm from FIrpic emission, combined with some leakage at short wavelength (also shown in the inset of Figure 3b) and exciplex emission at long wavelength, which both increase with increasing driving voltage. For the double emission peak of Firpic, one can also see a relative decrease at the shorter wavelength (474 nm) with increasing driving voltage, which comes from the interference effect due to the recombination shift toward the anode side. This also implies that the recombination zone takes place inside the OXD layer at lower driving voltage (6 V) and shifts toward the anode side with increasing driving voltage. When the dopant concentration further increases (6%–15% in devices 3 to 6, as shown in Figure 3c–f), the main recombination takes place in the highly efficient FIrpic dopants. Relative increase of longer wavelength peaks of Firpic emission with increasing driving voltages are observed for all the devices, which means the recombination zone shifts from inside the OXD toward the anode side. Besides, when comparing the EL spectra at low driving voltages (6 V) with different dopant concentrations (3%–15%), the relative intensity at shorter wavelength increases from 3% to 6%, and then decreases from 6 to 15%. Not only increasing electron injection capability, FIrpic also plays some role in hole injection and transport. Under low concentration (3%–6%), holes inject through the FIrpic molecules which results in a blueshift. With further increasing FIrpic concentrations, the redshift comes from better electron injection and transport, together with the retardation of the holes. Comparing the J-V curves in Figure 2a, the driving voltage is lowest for device 5 (12%), due to the better electron injection and transport. However, when further increasing the dopant concentration to 15%, an increase in driving voltage is observed which comes from the hole-trapping effect of FIrpic on the OXD matrix.

**Figure 4:**
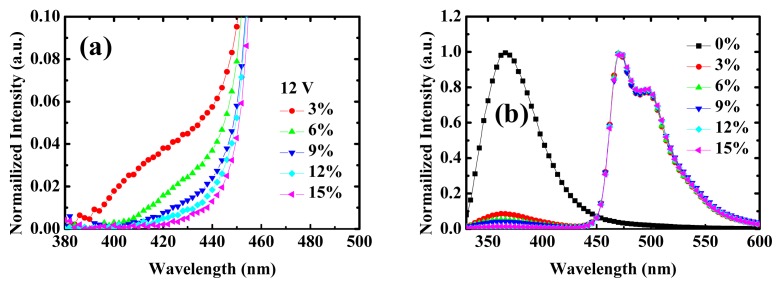
(**a**) Zoom-in of EL spectra of devices 1–6 at high voltages (12 V); (**b**) photoluminescence (PL) spectra of OXD thin film (100 nm) doped with different FIrpic concentrations (0%–15%).

Figure 4a shows the NPB emission at short wavelength (~430 nm) at 12 V for devices 2–6. One can see that the leakage decreases with increasing FIrpic concentration due to the increase of the recombination center. Figure 4b shows the photoluminescence (PL) spectra of OXD thin films doped with different concentrations (0%–15%). With increasing dopant concentration, emission from the OXD decreases which is transferred to the FIrpic emission. Besides, the emission spectra from FIrpic are always kept the same because the whole film is lit up with very little optical interference effect under optical pumping.

### 2.2. Device Performances of Blue Phosphorescent OLEDs with Different Doping Positions

**Figure 5:**
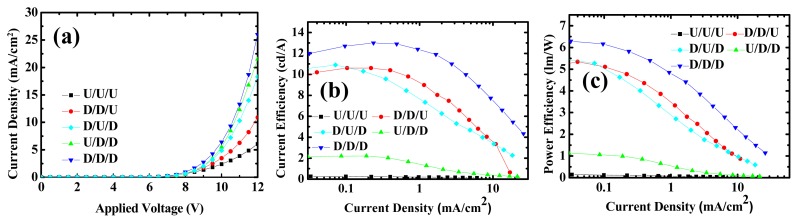
Comparison of (**a**) current density versus voltage; (**b**) current efficiency (cd/A) versus current density; and (**c**) power efficiency (lm/W) versus current density for devices 1, 4, 7, 8, and 9.

To further analyze the effects of FIrpic molecules on electrical and optical characteristics of OLEDs, we doped 9% FIrpic at different positions of the EML. As shown in Table 1, there is an intentionally undoped region next to ETL, at the center, and next to EBL for devices 7, 8, and 9, respectively. Figure 5a shows the J-V characteristics of devices 1, 4, 7, 8, and 9. J-V curves of selective doped OLED (devices 7, 8, and 9) are all within the range between undoped (device 1) and uniform-doped (device 4), because the dopants assist voltage reduction. For the OLED with the undoped region close to ETL (device 7), the driving voltage is significant higher, which means the FIrpic dopant plays an important role in facilitating electron injection. Comparing device 8 and device 4, a small voltage increase indicates better electron transport characteristics in the FIrpic doped OXD layer. As shown in Table 2, for device 9 with the undoped region close to EBL, the driving voltage (9.88 V) is quite close to that of the uniformly doped one (9.67 V). When the undoped layer is close to EBL (device 9), the hole trap (FIrpic) is removed which improves hole mobility. On the other hand, the electron mobility decreases. These two effects compete which results in the driving voltage of device 9 (9.88 V) being quite close to that of the uniformly doped one (9.67 V in device 4). It also implies that the voltage reduction phenomenon with incorporation of FIrpic into OXD does not result from the increase of the recombination current. Figure 5b shows the curves of current efficiency under different current density. When the undoped region is close to the EBL (device 9), the maximum efficiency decreases to 2.21 cd/A. This value is low because the main recombination locates near the interface of the EBL/EML. However, this value is not very low which means the recombination zone is broad and extends inside EML towards the ETL at least 10 nm, which explains that when the undoped region is at the middle and close to the ETL, the maximum efficiency is ~10 cd/A, which is still lower than that of device 4 (13 cd/A).

**Figure 6:**
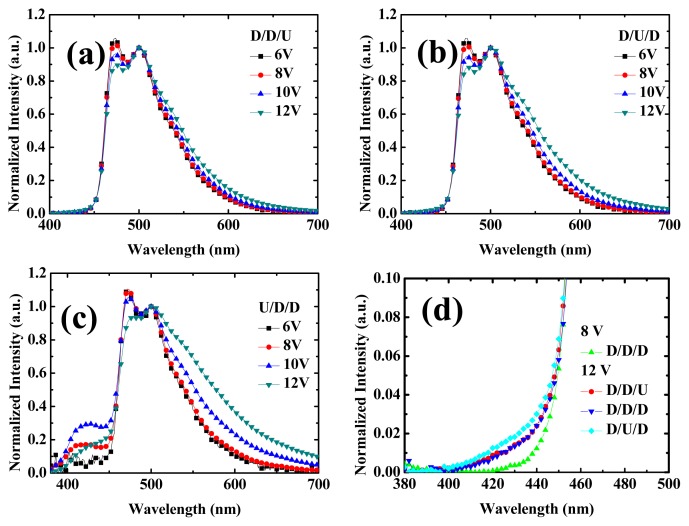
EL Spectra at different voltages of devices (**a**) 7, (**b**) 8, and (**c**) 9. (**d**) Zoom-in of EL spectra of devices 4, 7, and 8.

Figure 6a–c shows the EL spectra under different driving voltages of devices 7, 8 and 9, respectively. Figure 6a,b are nearly identical except the exciplex hump around 550 nm is larger for the case of device 8, when the undoped region is at the middle of the EML. Because FIrpic dopants act as the recombination center, when there is no dopant at the middle of the EML, more electrons may transport to the interface of the EBL/EML interface for exciplex emission. Figure 6d shows the leakage emissions from device 4, 7 and 8, respectively. NPB leakage is nearly identical for devices 4 and 7 respectively, because the whole recombination zone (20 nm close to the EBL) is doped with FIrpic. On the other hand, NPB leakage is slightly higher for the case of device 8 with undoped region at the middle of the EML. Some electrons penetrate into the HTL for emission. For the EL spectra of device 9 with the undoped region close to the EBL interface, the spectra peak at shorter wavelength (474 nm) is higher than that in device 7 and 8, because the recombination zone locates far away from the EBL/EML interface (at least 10 nm) which blue-shifts the spectrum. Observing the light leakage at short wavelength, one can see the NPB emission increases from 6, 8, and 10 V, then decreases at 12 V. This implies the electron penetrates inside the NPB, and may further transport into the anode without recombination. After all, NPB is also a good electron transporter. Hence, here we may deduce that FIrpic inside the OXD serves as a recombination center to confine carrier not penetrating into the EBL, as well as HTL.

## 3. Experimental Section

Our devices were fabricated on the patterned indium-tin-oxide (ITO) substrate with pixel size of 2 mm × 2 mm. After O_2_ plasma treatment, the device is transferred to the multisource evaporator for organic layer and cathode deposition under ultra high vacuum 5 × 10^−6^ torr. Then, it is transferred to the glovebox for the encapsulation process. Electrical and optical characteristics are determined with a source meter (Keithley 2400) and spectroradiometer (Minolta CS-1000), respectively. Photoluminescence (PL) of organic thin films is carried out by Hitachi F-4500.

## 4. Conclusions

In summary, by analyzing the J-V characteristics, efficiency, and EL spectra of OLEDs with different concentration and dopant profile of FIrpic in the OLED, we can conclude that: (1) FIrpic aids electron injection from ETL into EML; (2) it also helps electron transport; (3) on the other hand, under low dopant concentrations (3%–6%), it may also assist hole injection; (4) additionally, it is a hole trap which retards hole transport.

## Figures and Tables

**Table 1:** Layer structures of organic light-emitting devices (OLEDs).

Device	HTL	EBL	EML (30 nm)			ETL	EIL	Cathode

	NPB	mCP	FIrpic in OXD			BPhen	LiF	Al

1			0%					
2			3%					
3			6%					
4			9%					
5			12%					
6	50 nm	10 nm	15%			40 nm	1.2 nm	100 nm
						
**Device**			**EML1 (10 nm)**	**EML2 (10 nm)**	**EML3 (10 nm)**			
						
7			9%	9%	0%			
8			9%	0%	9%			
9			0%	9%	9%			

**Table 2:** Electrical and optical properties of the OLEDs.

Device	Volt. @5 mA/cm^2^	Max. lm/W	Max. cd/A
1	11.6	0.17 @ 5.5 V	0.77 @ 5 V
2	9.63	3.33 @ 6 V	6.38 @ 6.5 V
3	9.87	5.65 @ 6 V	11.4 @ 6.5 V
4	9.67	6.29 @ 6 V	13 @ 6.5 V
5	8.78	6.80 @ 6 V	14.2 @ 7 V
6	9.16	6.29 @ 6 V	14.1 @ 7.5 V
7	10.6	5.34 @ 6 V	10.6 @ 6.5 V
8	10	5.47 @ 6 V	10.9 @ 6.5 V
9	9.88	1.13 @ 6 V	2.21 @ 7 V
